# Clinical outcomes after mini-hook plate fixation for small avulsion fractures around the interphalangeal or metacarpophalangeal joints of the hand

**DOI:** 10.1186/s13018-021-02339-z

**Published:** 2021-03-12

**Authors:** Jung Il Lee, Ki-Chul Park, Hyun Soo So, Duk Hee Lee

**Affiliations:** 1 Department of Orthopedic Surgery, Korea University Guro Hospital, Seoul, South Korea; 2 Department of Orthopedic Surgery, Hanyang University Guri Hospital, Guri, South Korea; 3 Department of Emergency Medicine, Ewha Women’s University Mokdong Hospital, Seoul, South Korea

**Keywords:** Hook plate, Mini-hook plate, Mallet finger, Mallet fracture, Avulsion fracture, Phalangeal avulsion

## Abstract

**Background:**

Mini-hook plate has been described for the treatment of various small avulsion fragments in the hand. This retrospective study aimed to evaluate clinical outcomes after mini-hook plate fixation in patients with an avulsion fracture around the interphalangeal or metacarpophalangeal joints of the hand.

**Methods:**

Nineteen patients with avulsion fractures around the interphalangeal or metacarpophalangeal joints of the hand were included in this study. Seven patients had a mallet fracture, and 12 patients had other phalangeal avulsion fractures including central slip, collateral ligament, volar plate, and flexor avulsion fractures. The osseous union and functional outcomes, including finger joint motion, joint stability, pinching strength, and the disabilities of the arm, shoulder, and hand score, were evaluated.

**Results:**

The mean duration of follow-up was 33.8 months. All patients in mallet and other phalangeal avulsion fractures achieved osseous union between the avulsion fragment and phalangeal bone, and there was no joint subluxation. There were no significant differences in the disabilities of the arm, shoulder, and hand scores. However, the patients with mallet fracture have lower mean percentage values of the total active range of motion and pinching strength than other phalangeal avulsion fractures. We abandoned this procedure in mallet fractures because the early results after mini-hook plate fixation in mallet fractures appeared unfavorable.

**Conclusion:**

These results suggest that the mini-hook plate fixation can provide sufficient stability and good clinical outcomes in those with phalangeal avulsion fractures. However, the outcomes for mallet fractures were not as good as those for other phalangeal avulsion fractures.

## Backgrounds

Avulsion fracture around the interphalangeal or metacarpophalangeal joints of the hand contains tendons, collateral ligaments, and volar plate, which are critical structures that contribute to finger motion and stability. Accurate reduction and rigid fixation of avulsion fragments are mandatory to restore the function of these structures. The mini-hook plate is a useful method to fix small osseous fractures, and its use for phalangeal avulsion fractures was first described in mallet fractures [1, 2]. Clinical reports on the treatment of mallet fractures using the mini-hook plate showed that fixation is usually stable enough for early mobilization and excellent functional outcomes [3, 4]. Some surgeons extended the indication for the mini-hook plate fixation beyond mallet fracture to include other phalangeal avulsion fractures, including central slip of the extensor, collateral ligament, flexor tendons, and volar plate [5, 6]. They showed that this technique could be used for the treatment of other phalangeal avulsion fractures and gave good to excellent clinical results and enough stability to achieve early mobilization.

Although several studies showed that mini-hook plate fixation provides good surgical outcomes in mallet fractures, some authors also reported a relatively high complication rate with this technique for the treatment with mallet fractures [6, 7]. The complications reported included nail deformity, imminent perforation of the plate, and re-displacement of fracture fragment after hook plate fixation in mallet fractures.

After acknowledging positive clinical reports about this technique, we have been performing the mini-hook plate fixation technique to treat phalangeal avulsion fractures including mallet fractures and other phalangeal avulsion fractures. This study aimed to evaluate the clinical outcomes of mini-hook plate fixation in patients with mallet fractures and other phalangeal avulsion fractures.

## Methods

**Fig. 1:**
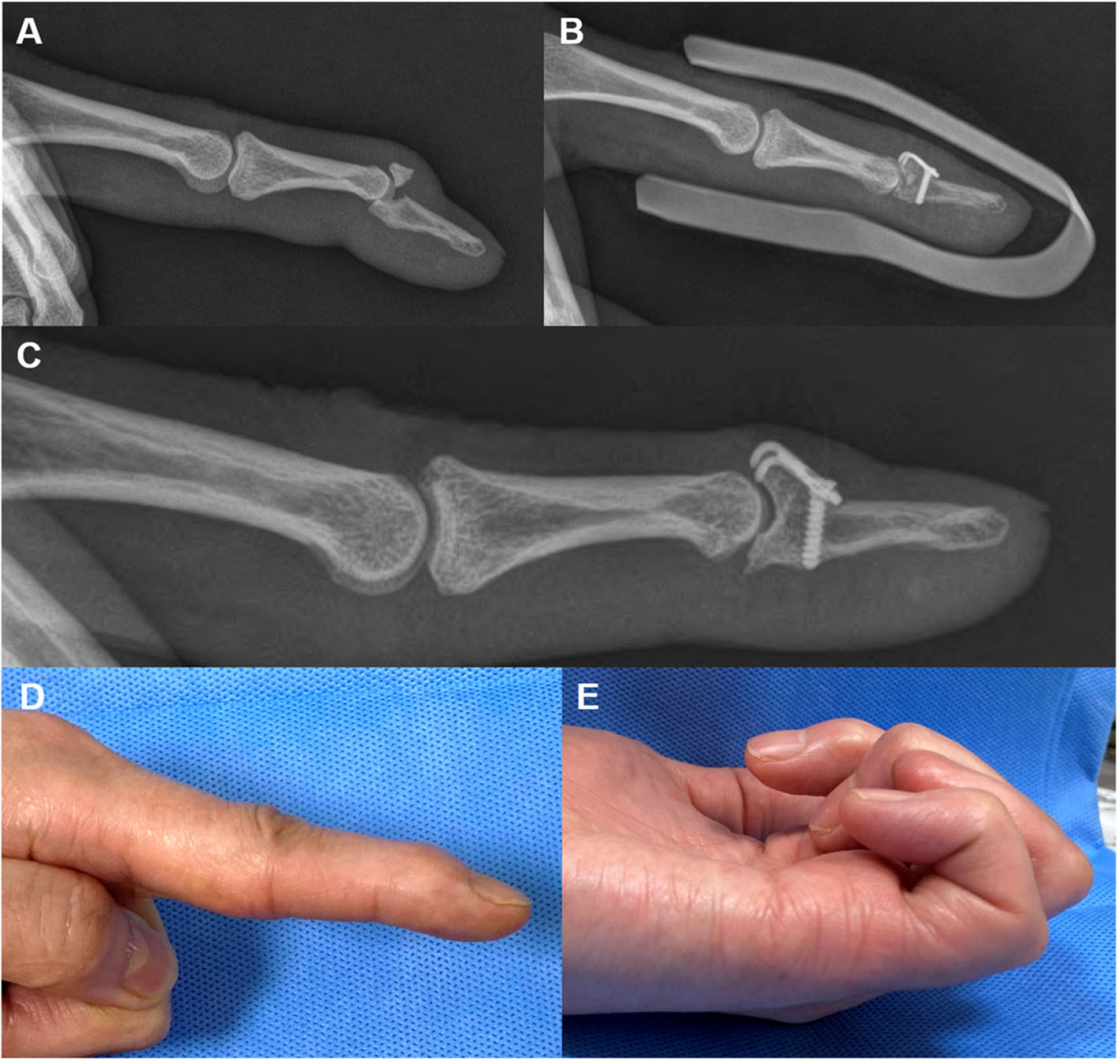
Mallet fracture (**a**). The mini-hook plate captured the dorsal avulsed fragment, and the joint was reduced (**b**). Plain radiography 5 years post-operation revealing that osseous union, no metal failure, and no subluxation of the joint. However, an irregular articular surface is visible (**c**). The total range of DIP joint motion was 69%, and the pinching strength was 43% compared to the values of the same contralateral joint (**d**, **e**)

**Fig. 2:**
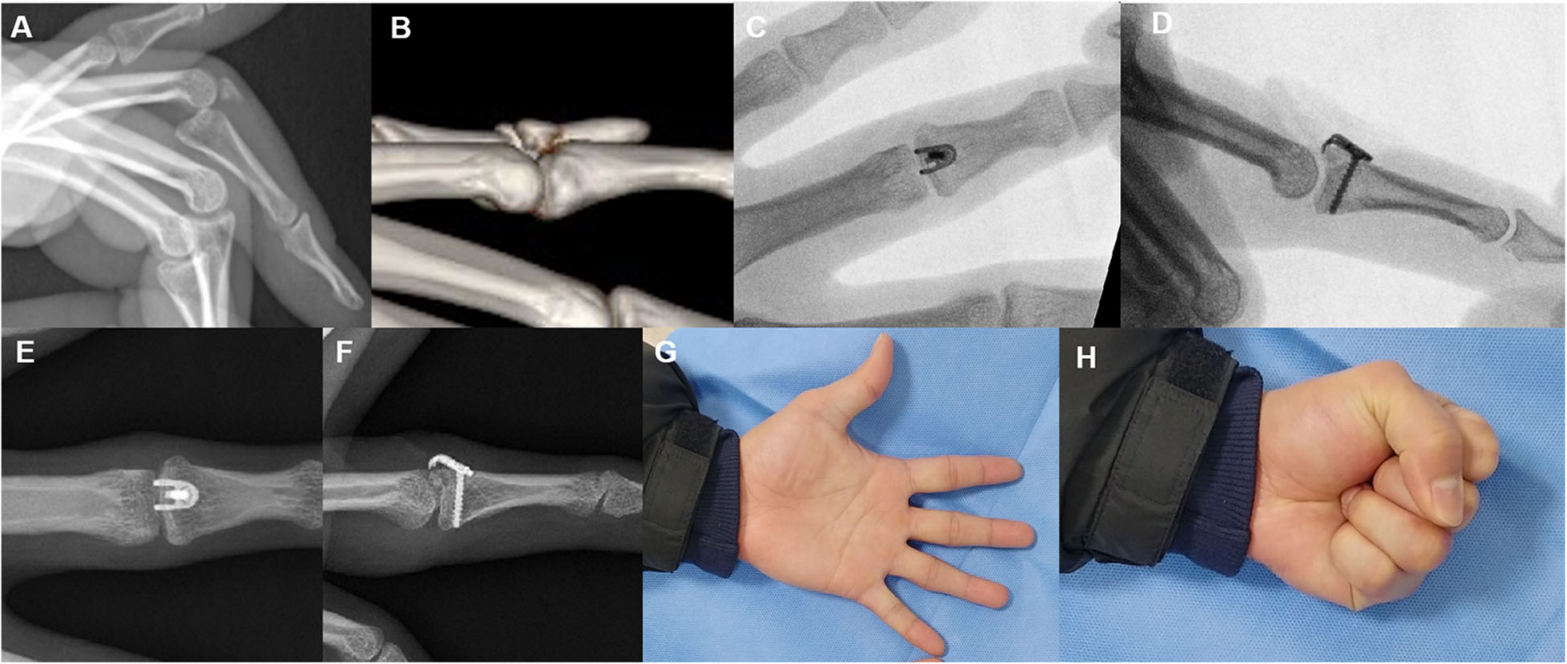
Central slip avulsion fracture with volar subluxation of PIP joint (**a**, **b**). The mini-hook plate captured the dorsal avulsed fragment, and the joint was reduced (**c**, **d**). Plain radiography 3 years post-operation revealing that osseous fragment was well united and that there was no metal failure or joint subluxation (**e**, **f**). The total range of PIP joint motion was 95%, and the pinching strength was 90% compared to the values of the same contralateral joint (**g**, **h**)

**Fig. 3:**
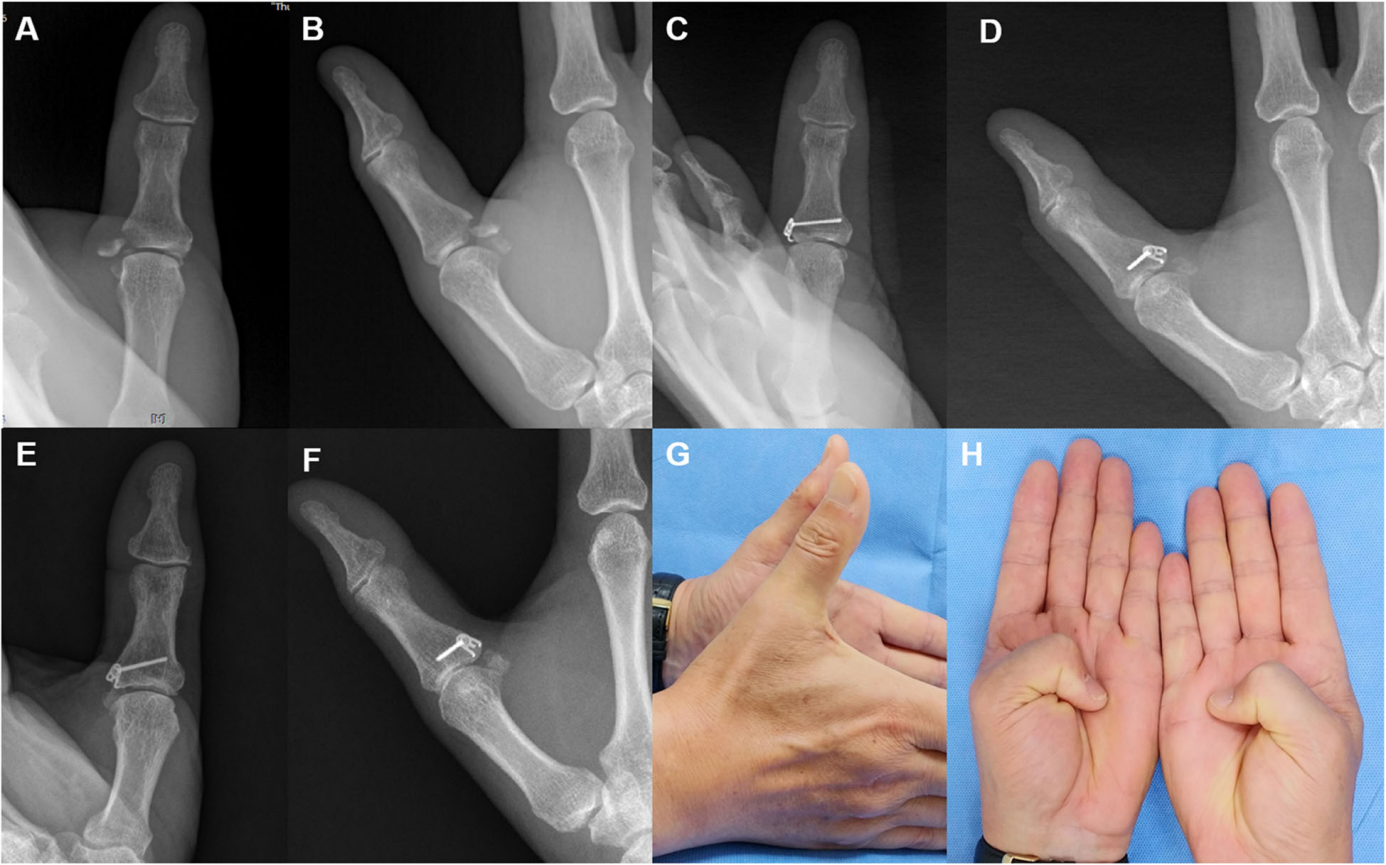
Ulnar collateral ligament avulsion fracture (**a**, **b**). The avulsed fragment was reduced and captured by the mini-hook plate (**c**, **d**). Plain radiography 55 months post-operation revealing that the osseous fragment was well united (**e**, **f**). The total range of MP joint motion was 82%, and the pinching strength was 98% compared to the values of the same contralateral joint (**g**, **h**)

**Fig. 4:**
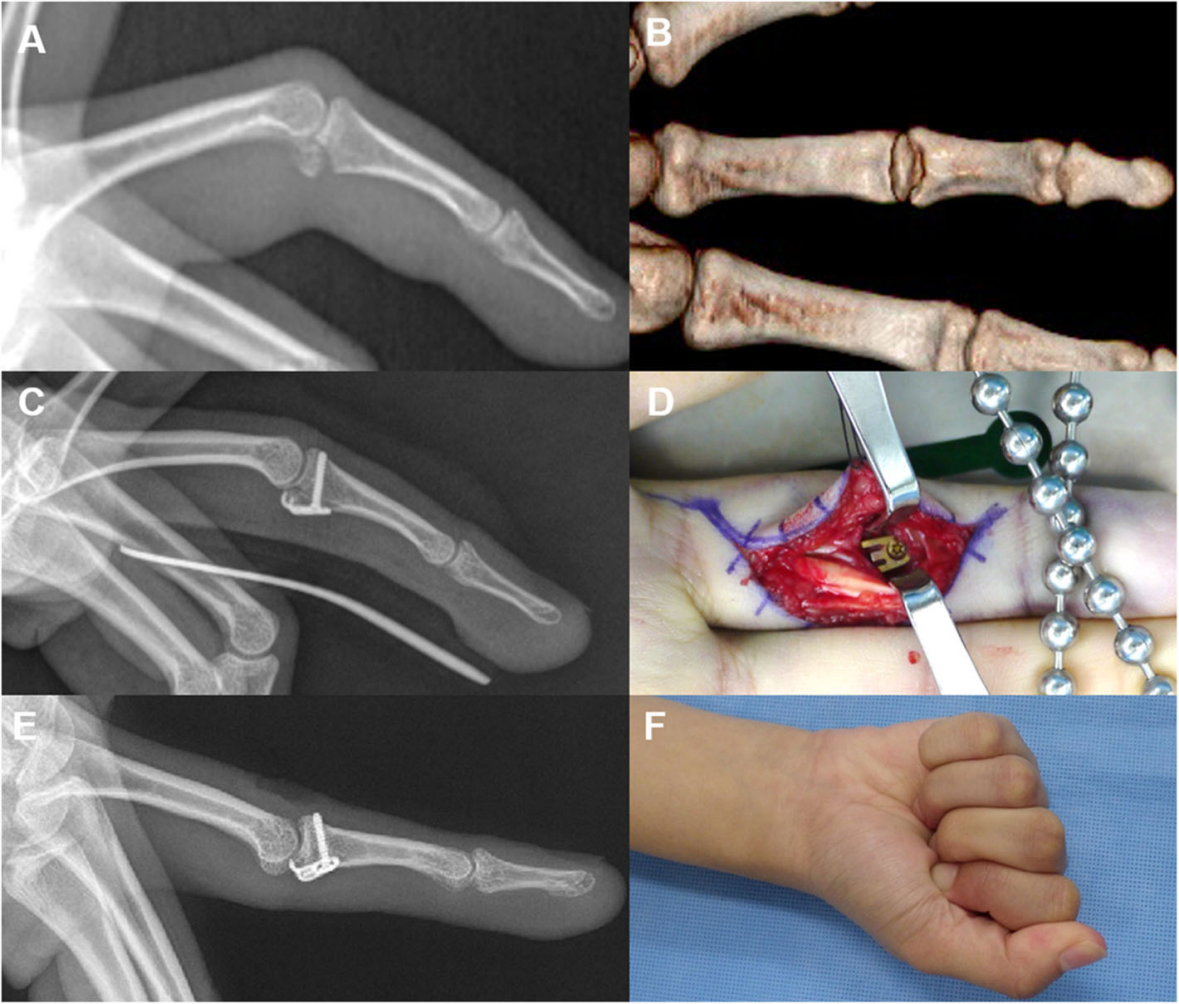
Volar plate avulsion fracture (**a**, **b**). The fragment was reduced, and a hook plate was placed over the fracture fragment, with the proximal hook end capturing the soft volar plate after opening the A3, C2, and C3 pulleys and then retracting the deep and superficial flexors (**c**, **d**). Plain radiography 55 months post-operation revealing that the osseous fragment was well united. The total range of MP joint motion was 100%, and the pinching strength was 90% compared to the values of the same contralateral joint (**e**, **f**)

**Fig. 5:**
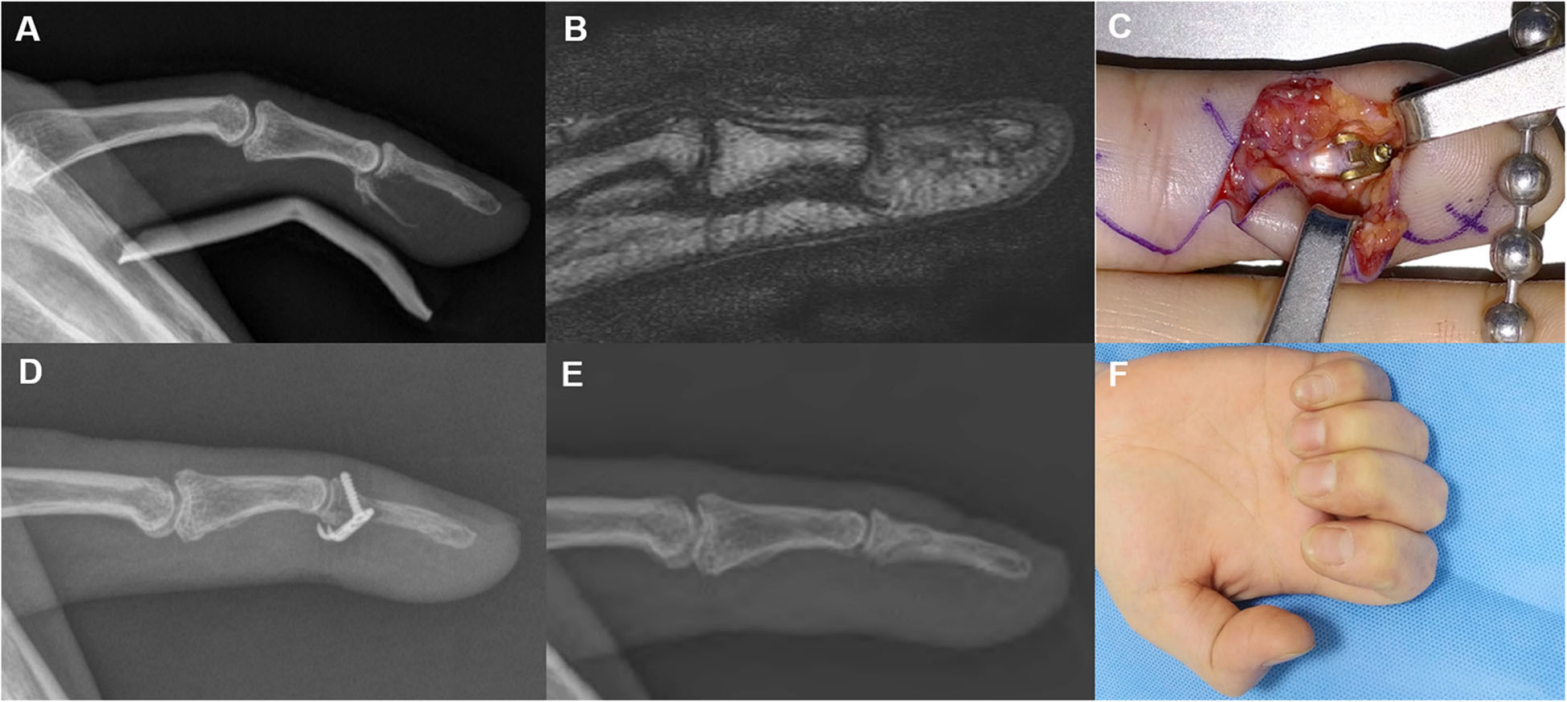
Flexor digitorum profundus (FDP) avulsion fracture (**a**, **b**). A mini-hook plate captured the avulsed fragment with FDP (**c**, **d**). The patient underwent plate removal and plain radiography 6 months post-operation reveals that the osseous fragment was well united and that there was no joint subluxation. The total range of DIP joint motion was 80%, and the pinching strength was 90% compared to the values of the same contralateral joint (**e**, **f**)

We retrospectively enrolled patients in this study who underwent open reduction and internal fixation using a 1.2-mm mini-hook plate (Medartis, Basel, Switzerland) between August 2014 and November 2019. The study was approved by our institutional review board (2020-06-021). Nineteen patients with phalangeal avulsion fractures were included in this study. Seven patients had a mallet fracture of the distal phalanx (Fig. 1). Four patients had a dorsal avulsion fracture of the extensor central slip from the middle phalanx (Fig. 2). Three patients had a phalangeal collateral ligament avulsion fracture. Three patients had ulnar collateral ligament avulsion fracture of the thumb (Fig. 3). One patient had an avulsion fracture of the volar plate from the middle phalanx (Fig. 4). One patient had a flexor digitorum profundus (FDP) avulsion from the distal phalanx (Fig. 5).

All patients were assessed by plain radiography, including anteroposterior, lateral, and oblique views, and computerized tomography (CT) before surgery to determine the displacement, size, and degree of comminution of the avulsion fragment. Operative treatment using the mini-hook plate fixation technique for mallet fracture was indicated if the avulsion fragment involved at least one third of the intra-articular surface, and volar subluxation of the distal phalanx was noted. Operative fixation using a mini-hook plate in other phalangeal avulsion fractures was indicated if the avulsion fracture fragment was unstable and displaced over 2 mm, which caused joint instability and loss of function of the avulsed tendon or ligament.

One orthopedic hand surgeon performed all surgical procedures. After plate fixation, the stability was evaluated by moving the interphalangeal (IP) or metacarpalphangeal (MCP) joint passively into full flexion under fluoroscopy. The joint was immobilized in full extension in a thermoplastic splint for 2 weeks. Active/passive motions were started 2 weeks after surgery. For FDP avulsion fracture, the patient followed the flexor tendon rehabilitation protocol for 6 weeks.

### Outcome measurements

The osseous union based on plain radiographs and functional outcomes, including finger joint motion, joint stability, pinching strength, and the disabilities of the arm, shoulder, and hand (DASH) score, were evaluated at the final follow-up. Osseous union was judged by bridging trabeculae at the fracture site. The active ranges of motion of the proximal interphalangeal (PIP) or distal interphalangeal (DIP) joints were measured with a finger goniometer in the affected and non-affected digits. The stability of the reconstructed joint was investigated in varus and valgus, stressing the joint in extension and 30° of flexion. The pinching strength was measured using a hydraulic pinch gage (Fabrication Enterprises, White Plains, NY, USA) and recorded a mean value of three trial values. Fellowship-trained hand surgeons evaluated finger motion, stability, and pinching strength. Measured values were presented as a percentage of those in the contralateral uninjured digits. The DASH questionnaire (range, 0-100, with 0 as the best result) was self-administered by the patients. All values were presented as the mean value with a standard error of the mean. Statistical analysis was performed using the Mann-Whitney *U* test to compare outcomes of mallet fractures and other phalangeal avulsion fractures. A *P* value of less than 0.05 was considered statistically significant.

## Results

**Fig. 6:**
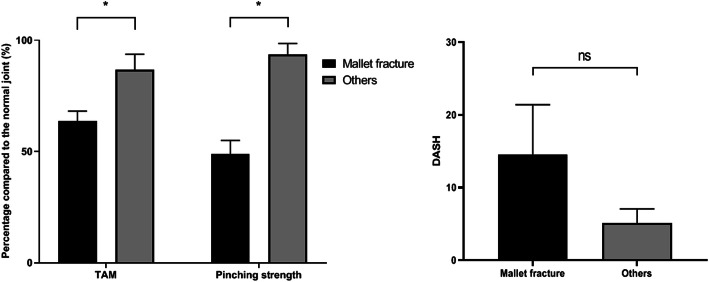
Total active range of motion (TAM) and pinching strength in those with other phalangeal avulsion fractures (others) were better than in those with mallet fractures. The disabilities of the arm, shoulder, and hand (DASH) score for other phalangeal fractures was lower than that for mallet fractures, but this difference was not significant. (*P < 0.05; ns, no significance; the values of clinical outcomes are presented with percentage values compared to the same contralateral joint)

The mean duration of follow-up was 33.8 months (range 6-60). All patients achieved osseous union between the avulsion fragment and phalangeal bone, and there was no joint subluxation on the last follow-up plain radiographs. All patients returned to their pre-injury levels of activity and occupations. However, the patients with mallet fracture treated by mini-hook plate fixation were less satisfied than other phalangeal fractures, because the patients with mallet fracture had residual mallet deformity, limited active range of motion, and decreased pinching strength even though radiologic good outcomes. The mean percentage value of the total active range of motion (TAM) of the involved joint compared to that of the contralateral uninjured same joint in other phalangeal avulsion fractures was 86.5±6.6%, which was significantly better than the mean value (63.5±4%) for mallet fractures (Fig. 6). Physical examination with varus/valgus stress revealed that all involved joints had good stability. The mean percentage value of pinching strength in other phalangeal avulsion fractures was 93.5±4.7%, which was significantly better than the mean value (48.8±4.9%) obtained for mallet fractures. The mean DASH scores in other phalangeal avulsion fractures and mallet fractures were 3.1±1.7 and 18.0±11.0, respectively. However, the difference was not significantly different.

**Fig. 7:**
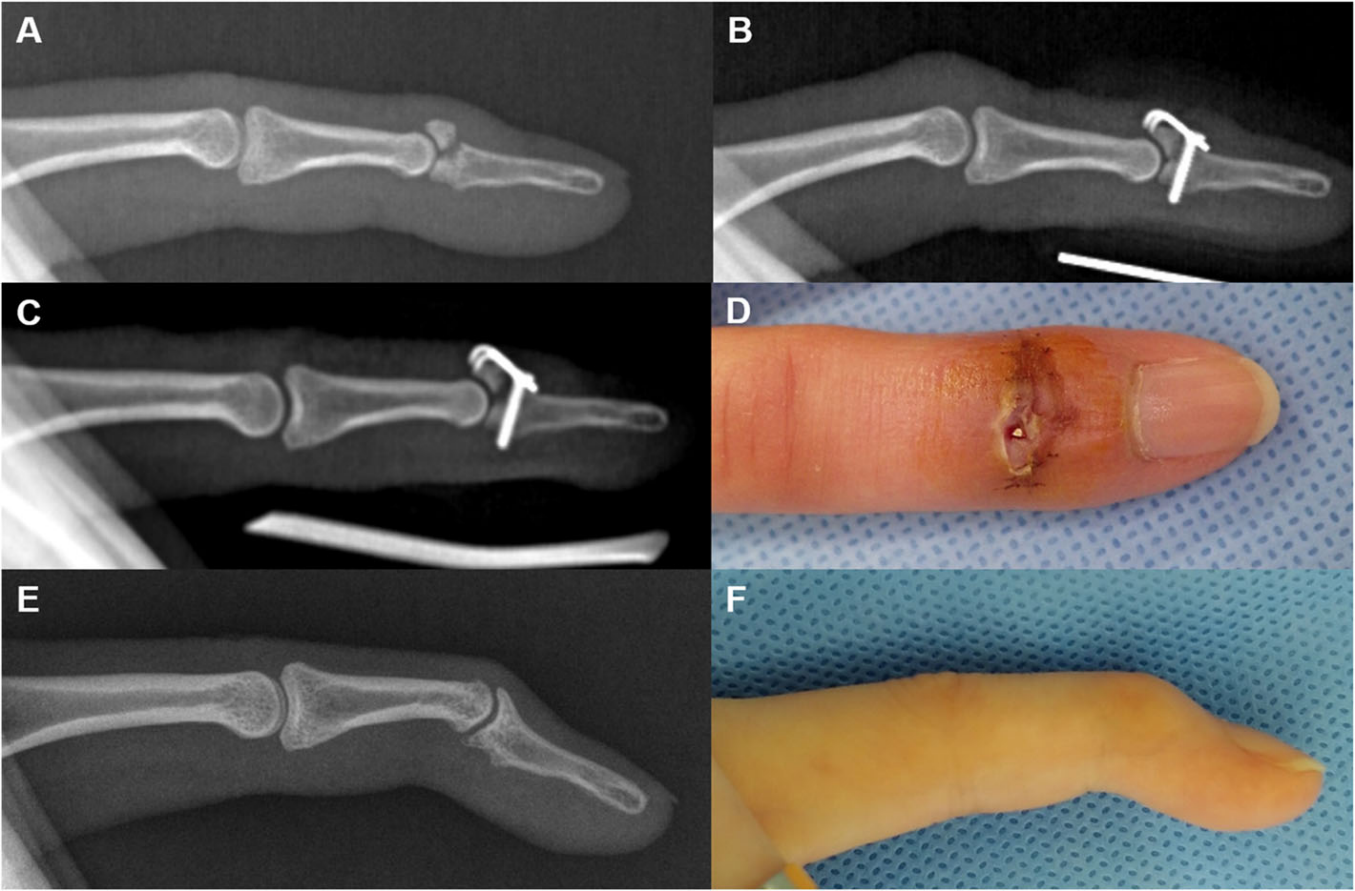
Mallet fracture (**a**). A mini-hook plate captured the dorsal avulsed fragment (**b**). Plain radiography 2-weeks post-operation demonstrated that the mallet fragment with the hook plate had slightly migrated proximally and dorsally (**c**). The plate was exposed to the dorsal skin, and the patient underwent revision surgery (extension block pinning) after plate removal (**d**). Plain radiography 57 months post-operation reveals that the osseous fragment was well united, but the articular surface was incongruent. The total range of DIP joint motion was 50%, and the pinching strength was 45% compared to the values of the same contralateral joint (**e**, **f**)

We generally do not recommend the plate removal, unless there are plate-related complications, such as skin or soft tissue irritation, tendon adhesion, or local tenderness. Among patients with other phalangeal avulsion fractures, three patients without complications underwent requested plate removal upon their request. Among patients with mallet fractures, three patients reported plate-related complications, and their plates were removed. One patient with mallet fracture had dorsal skin ulceration and metal plate exposure 2 weeks after surgery that was caused by a slight pull-out of the plate (Fig. 7). The patient underwent revision surgery and extension block pinning after the plate removal. However, plain radiographs 5 years after surgery showed an irregular articular surface of the DIP joint. The patient had 50% of TAM and 43% of pinching strength compared to that on the contralateral side for the same joint.

## Discussion

There are several other surgical techniques described for phalangeal avulsion fractures, such as Kirschner’s wires fixation, percutaneous extension block pinning [8], small interfragmentary screws [9], ligament or tendon repair with fragment excision [10], and tension band wiring [11]. However, each of these techniques has some limitations for avulsion fractures. The mini-hook plate can overcome these shortcomings of the former fixation techniques because this plate acts as both a buttress and tension band, without the placement of a screw or Kirscher’s wire across the small avulsed osseous fragment [5]. A previous cadaveric biomechanical study comparing the mini-hook plate and single suture anchor for fixing the avulsion fragment of the thumb ulnar collateral ligament demonstrated that the plate construct was biomechanically superior to the suture anchor construct [12]. The fixation is usually stable enough for early joint motion. Some surgeons state that the mini-hook plate can achieve the operation goals, which are anatomical reduction, rigid internal fixation, and early mobilization without the risk of fragmentation of the small osseous fragment [2, 4].

Although an initial report of the mini-hook plate fixation for the treatment of mallet fractures showed reasonable outcomes, the high incidence of complications was noteworthy. Early investigators reported no complications after the mini-hook plate fixation for mallet fractures [1, 2]. Szalay et al. showed that the mini-hook plate could provide very good or good functional outcomes. However, they reported a 15% (9/59 patients) complication rate with seven nail growth defects and two skin perforations caused by the plate [13]. Tie et al. fixed 31 mallet fractures with a mini-hook plate and reported a 22% complication rate with one nail deformity, three transient skin flap ischemia, and three fracture re-displacements [7]. They concluded that this technique still has value, with outcomes and complication rates comparable to the other treatment modalities. Nevertheless, the functional outcomes of their case series were not as excellent as initially reported.

Some surgeons extended indications for the mini-hook plate fixation beyond mallet fracture [5, 6, 14, 15]. They used the mini-hook plate for the treatment of other phalangeal avulsion fractures, such as a central slip of extensor, collateral ligament, flexor tendons, and volar plate avulsion fracture. All reports to treat other phalangeal avulsion fractures using the mini-hook plate showed no biomechanical failures and good functional outcomes. Our results of other phalangeal avulsion fractures, except mallet fracture, are in constituency with their reports. The mini-hook plate fixation is a versatile and effective method to permit early joint motion and good functional outcomes in small phalangeal avulsion fractures [5]. Thirumalai et al. reported that the mini-hook plates can cause tendon adhesions and joint stiffness in the case of dorsal fracture-dislocations of the PIP joint. However, PIP joint motion limitation is common after open reduction and plate fixation in PIP joint fracture-dislocation [6, 16]. Nevertheless, caution should be exercised in extending our results for PIP joint fracture-dislocation because this case series did not include cases of the PIP joint fracture-dislocation where mini-hook plate fixation was employed.

Our study investigated the functional outcomes after mini-hook plate fixation in both mallet fractures and other phalangeal avulsion fractures and compared them. The previous two studies did not compare the functional outcomes between mallet fracture and other phalangeal avulsion fractures. However, they implied that this technique is more suitable for treating phalangeal avulsion fractures other than mallet fractures [6, 15]. Mehling et al. treated 36 fractures, including 24 mallet fractures and 11 other phalangeal avulsion fractures. They reported seven complicated patients among 24 patients with mallet fractures, with five nail growth defects, one infection, and one secondary dislocation of the implant. However, there were no complications, and good outcomes were observed in other phalangeal avulsion fractures. Thirumalai et al. reported 11 complicated patients among 35 patients with mallet fractures, with six nail deformities and five plate extrusions. They concluded that mini-hook plate fixation was a versatile technique for the fixation of phalangeal avulsion fractures. However, complication rates can be high when it is used for mallet fractures. There were three complications in our series of mallet fractures: two plate irritations and one plate exposure with post-traumatic arthritis (Fig. 7). We initially tried to perform mini-hook plate fixation in all cases of phalangeal avulsion fractures. However, the clinical results after mini-hook plate fixation in mallet fractures appeared unfavorable. Subsequently, we abandoned this procedure and switched to extension block pinning for the treatment of mallet fractures. Intraoperatively, the surgical procedures in mallet fractures were technically demanding and time-consuming, because of not enough wide space in the distal phalanx for proper positioning of plate avoiding nail matrix injury. Moreover, this issue should be more considered in female patients with ring and little finger mallet fractures. Early studies also noted the shortcomings of mini-hook plate fixation in mallet fractures, which is technically demanding, higher cost than extension block pinning, and may need secondary surgery for plate removal [3, 4].

This study has several limitations that require consideration. First, the study was a retrospective comparative case series with a small sample size. Previous investigations [3–7, 13, 17] of mini-hook plate fixation were also retrospective case studies because the incidence of phalangeal avulsion fractures is relatively low, and there was insufficient evidence for planning a prospective study. A larger-scale, prospective study is necessary to support our results and obtain more outcome data. Second, we categorized various fracture types of phalangeal avulsion fracture except for mallet fracture into others. Previous investigators also categorized by the same manner, which comes from the low incidence of each type of phalangeal avulsion fractures [5, 6]. However, the clinical outcomes in other phalangeal avulsion fractures should be carefully interpreted. To overcome this shortcoming, we measured the objective outcomes such as range of motion as a percentage of the same joint in the contralateral uninjured digits. Third, it is possible that the operator might not reach the plateau of the learning curve for mini-hook plate fixation in mallet fractures. We abandoned this procedure for mallet fractures after observing poor clinical outcomes in the first seven patients. This might have contributed to poor clinical outcomes in our case series of mallet fractures. However, the initial six patients with other phalangeal avulsion fractures were treated in the same period in which mini-hook plate fixation was performed for mallet fractures. Despite the learning curve being the same, patients with other phalangeal avulsion fractures showed excellent functional outcomes.

## Conclusions

Our results suggest that mini-hook plate fixation can provide acceptable clinical outcomes including restoration of joint motion, stability, pinching power, and self-reported clinical scores in patients with phalangeal avulsion fractures. However, in the case of mallet fractures, this technique might be unable to provide acceptable surgical outcomes, unlike in cases of other phalangeal avulsion fractures. Moreover, it might lead to plate-related complications, such as plate extrusion, skin irritation, and nail deformity. It is essential to ensure that the surgical techniques, including handling of small osseous fragments and placement of the hook plate in the accurate position, are meticulous.

## Data Availability

All data generated or analyzed during this study are included in this published article.
